# Alternative splicing of *ZmCCA1* mediates drought response in tropical maize

**DOI:** 10.1371/journal.pone.0211623

**Published:** 2019-01-30

**Authors:** Lei Tian, Xiyong Zhao, Haohao Liu, Lixia Ku, Shunxi Wang, Zanping Han, Liancheng Wu, Yong Shi, Xiaoheng Song, Yanhui Chen

**Affiliations:** 1 College of Agronomy, National Key Laboratory of wheat and Maize Crop Science, Henan Agricultural University, Zhengzhou, China; 2 Crop Research Institute, Peanut Research Laboratory, Anhui Academy of Agricultural Sciences, Hefei, China; 3 College of Agronomy, Henan University of Science and Technology, Luoyang, China; National Taiwan University, TAIWAN

## Abstract

The circadian clock regulates numerous biological processes in plants, especially development and stress responses. *CIRCADIAN CLOCK-ASSOCIATED 1* (*CCA1*) is one of the core components of the day–night rhythm response and is reportedly associated with ambient temperature in *Arabidopsis thaliana*. However, it remains unknown if alternative splicing of *ZmCCA1* is modulated by external stress in maize, such as drought stress and photoperiod. Here, we identified three *ZmCCA1* splice variants in the tropical maize line CML288, which are predicted to encode three different protein isoforms, i.e., ZmCCA1.1, ZmCCA1.2, and ZmCCA1.3, which all retain the MYB domain. In maize, the expression levels of *ZmCCA1* splice variants were influenced by photoperiod, tissue type, and drought stress. In transgenic *A*. *thaliana*, *ZmCCA1*.*1* may be more effective than *ZmCCA1*.*3* in increasing drought tolerance while *ZmCCA1*.*2* may have only a small effect on tolerance to drought stress. Additionally, although *CCA1* genes have been found in many plant species, alternative *CCA1* splicing events are known to occur in species-specific ways. Our study provides new sight to explore the function of *ZmCCA1* splice variants’ response to abiotic stress, and clarify the linkage between circadian clock and environmental stress in maize.

## Introduction

Subtropical and tropical maize lines have been used as germplasm to improve maize quality and yield compared with temperate maize lines due to their abundant genetic variations. However, photoperiod sensitivity in maize restricts the utilization of subtropical and tropical germplasms under long day (LD) conditions because of a delayed floral transition [1, 2]. The circadian clock is involved in photoperiod-mediated flowering and accurately perceives external input signals to generate endogenous rhythmic outputs during an approximate 24 h cycle, which can be synchronized with the environment by regulating key basic metabolic processes including photosynthesis, hypocotyl elongation, and floral transition [3]. Moreover, the clock can also regulate the stomatal aperture, rhythmic leaf movement, and roots and stem circumnutating in plants [4, 5].

In recent years, the transcriptional regulation and molecular functions of circadian clock genes have been studied in *Arabidopsis thaliana*, including *CCA1* [6], *LATE ELONGATED HYPOCOTYL* (*LHY*) [7], *TIMING OF CAB EXPRESSION 1* (*TOC1*), and *GIGANTEA* (*GI*). Feedback loops mediated by *CCA1*, *LHY*, and *TOC1* constitute one of the circadian clock regulatory models whereby CCA1 and LHY can bind directly to the *TOC1* promoter as the transcriptional repressors that suppress TOC1 accumulation. Conversely, TOC1 represses the expression of *CCA1* and *LHY* through directly binding their promoters [8]. However, the molecular mechanisms of the circadian clock gene in maize are yet to be reported; therefore, cloning of these associated genes will provide a theoretical foundation for understanding the maize circadian clock.

Alternative splicing (AS) events are always associated with growth, signal transduction, circadian rhythms, and abiotic stresses in plants [9]. In moss, 1779 AS events identified by genome-wide analysis and accounting for nearly half of the expressed genes showed significant responses to high-temperature stress [10]. Additionally, approximately 7500 genes identified in tomato pollen demonstrated heat-dependent accumulation of intron retention and exon skipping, including six heat-shock factors and 29 heat-shock proteins that play important roles in plant responses to heat stress [11]. Furthermore, AS phenomena of some genes related to the *A*. *thaliana* circadian clock are reportedly involved in defense responses [12]. For example, the *SNW/Ski-interacting protein* gene, coding a SNW/Ski-interacting protein component of the spliceosome, is regulated by salt, mannitol, and abscisic acid (ABA) treatment. It serves as a major link between the circadian clock and AS under biotic stress conditions [13]. Moreover, two splice variants of *CCA1*, *CCA1α* and *CCA1β*, were shown to be modulated by low temperature in *A*. *thaliana* [14]. However, only limited studies are available regarding the function of maize *ZmCCA1* splice variants under other environmental stresses, such as drought.

In this study, we cloned *ZmCCA1* from the tropical maize line CML288 using homologous cloning and the 3′RACE technique and identified three splice variants encoding three protein isoforms. We systematically investigated the expression of *ZmCCA1* splice variants in leaves and leaf sheaths of CML288 plants at five fully expanded-leaf stage under various environmental conditions. Compared with wild-type (WT) plants, transgenic *A*. *thaliana* plants overexpressing *ZmCCA1*.*1* and *ZmCCA1*.*3* obviously showed higher tolerance to drought stress while transgenic *A*. *thaliana* plants overexpressing *ZmCCA1*.*2* exhibited slightly enhanced drought tolerance. Cross-species examination suggests that AS events of *CCA1* are present in numerous species, but occur with species specificity. Our data will help clarify the linkage between circadian clock and environmental stress in maize. Moreover, the AS of *ZmCCA1* should play more complex roles than previously expected.

## Materials and methods

The maize inbred line CML288 used in this study was collected from the International Maize and Wheat Improvement Center (CIMMYT) in Mexico. All *A*. *thaliana* were Columbia-0 (Col-0) unless otherwise specified.

Total RNA of leaves was isolated from CML288 five-leaf seedlings in a controlled culture room under LD conditions (16-h light/8-h dark) at 28°C with the TRIzol reagent (Invitrogen, Carlsbad, CA, USA). For 3′RACE, the first-strand cDNA was synthesized via the 3′-Full RACE Core Set kit (v. 2.0; Takara Bio, Dalian, China). For homologous cloning, first-strand cDNA was synthesized via the PrimeScript 1st Strand cDNA Synthesis Kit (Takara Bio). Based on the cDNA sequence of *ZmCCA1* (GenBank number: EU954568.1), 3′RACE sequencing results, and gDNA sequence (GenBank number: AC215881.5), the primers were designed using Primer Premier 6 software (S1 Table).

The sequence analysis and multiple sequence alignments were analyzed by DNAMAN (v. 9.0; Lynnon Biosoft, San Ramon, CA, USA). The open reading frame (ORF) was acquired by the ORF Finder tool (https://www.ncbi.nlm.nih.gov/orffinder/). Molecular weight and isoelectric point (pI) were predicted by ExPASy (http://www.expasy.org/). Domain prediction was performed by Conserved Domain Search Service (CD Search) and SMART software (http://smart.embl-heidelberg.de/). The phylogenetic tree was constructed by MEGA 6.0 software (http://www.megasoftware.net/) using the maximum-likelihood method.

To produce transgenic *A*. *thaliana* lines with overexpression of *ZmCCA1*.*1*, *ZmCCA1*.*2*, and *ZmCCA1*.*3*, the entire ORF of these splice variants were subcloned into the *pCAMBIA1304* vector under control of the *CaMV 35S* promoter, respectively. *Agrobacterium tumefaciens*-mediated *A*. *thaliana* transformation was performed according to a modified floral dip method [15]. Homozygotic lines were obtained by hygromycin selection and analysis of segregation ratios, and T4 homozygotic seeds were used for the stress treatment.

CML288 seeds were grown in a controlled culture room at 28°C under short day (SD) conditions (8-h light/6-h dark) or LD conditions (16-h light/8-h dark). At the five fully expanded leaf stage, the leaves and leaf sheaths of the fifth leaf obtained from the same seedling were harvested over a 24-h period (ZT0–ZT8), starting at Zeitgeber time 0 (ZT0) with 3 h intervals between harvests, then immediately stored at −80°C until use. Three biological replicates were performed in this experiment, and three seedlings were mixed per biological replicate.

CML288 seeds were grown in vermiculite in a controlled culture room under SD conditions (28°C 8-h light/22°C 16-h dark). Then the grown uniform seedlings at two fully expanded leaf stage were then transferred to the pots with 2-L full-strength Hoagland’s nutrient solution. At the five fully expanded leaf stage, seedlings were treated with 20% (w/v) polyethylene glycol (PEG) 6000 before light irradiation under 24-h light conditions [16, 17]. Leaves (the fifth leaf) and their leaf sheaths were harvested every 3 h after 20% PEG-6000 treatment over a 24-h period (ZT0–ZT8), starting at ZT0, and immediately stored at −80°C until use. Three biological replicates were collected at each time point. Each tissue sample contained three different randomly uniform selected plants.

Relative water contents (RWCs) were measured under drought stress conditions compared to that under normal conditions according to our previous report [17]. Two days after drought stress treatment, the RWC was calculated using the following formula: RWC (%) = [(FM − DM)/(TM − DM)] × 100, where FM, DM, and TM refer to the fresh, dry, and turgid masses of the tissue, respectively [17].

As described in previous reports with minor modification [18, 19], the seeds of WT *A*. *thaliana* and transgenic T4 lines were surface sterilized with 5% NaClO for 5 min and washed five times with sterile distilled water. Approximately 50 seeds were placed on 1/2 MS medium with 0, 50, 150, 250 mmol/L mannitol with three replicates and incubated at 4°C in the dark for 2 days. Then the medium plates were then vertically cultured at 20°C for 2 weeks under LD conditions (16-h light/8-h dark). Germination (emergence of radicle) was calculated daily for 7 days after transferring to LDs. The length of the primary root was investigated after 2 weeks.

Total RNAs were extracted using TRIzol reagent according to the manufacturer’s instructions (Invitrogen), and first-strand cDNA was prepared by using HiScript Q RT SuperMix for qPCR with gDNA Eraser as per the manufacturer’s instructions (Vazyme, Nanjing, China). Quantitative real-time RT-PCR (qRT-PCR) was conducted on a Bio-Rad iQ5 Real-Time PCR System (Bio Rad Laboratories, Richmond, CA, USA). Each PCR reaction mix consisted of 1.0 μL diluted cDNA, 0.5 μL forward/reverse primers (10 μM), 12.5 μL 2 × Taq Plus Master Mix (Vazyme), 1.5 μL 10 × SYBR Green I (Invitrogen), and 9.0 μL RNase-free water in a total volume of 25 μL. Relative expression levels of the target genes were analyzed using the 2^−ΔΔCt^ method [20]. The 18s rRNA gene was used as an endogenous reference, and all experiments were performed using three biological replicates.

## Results

### Cloning and sequence analysis of *ZmCCA1*

**Fig 1:**
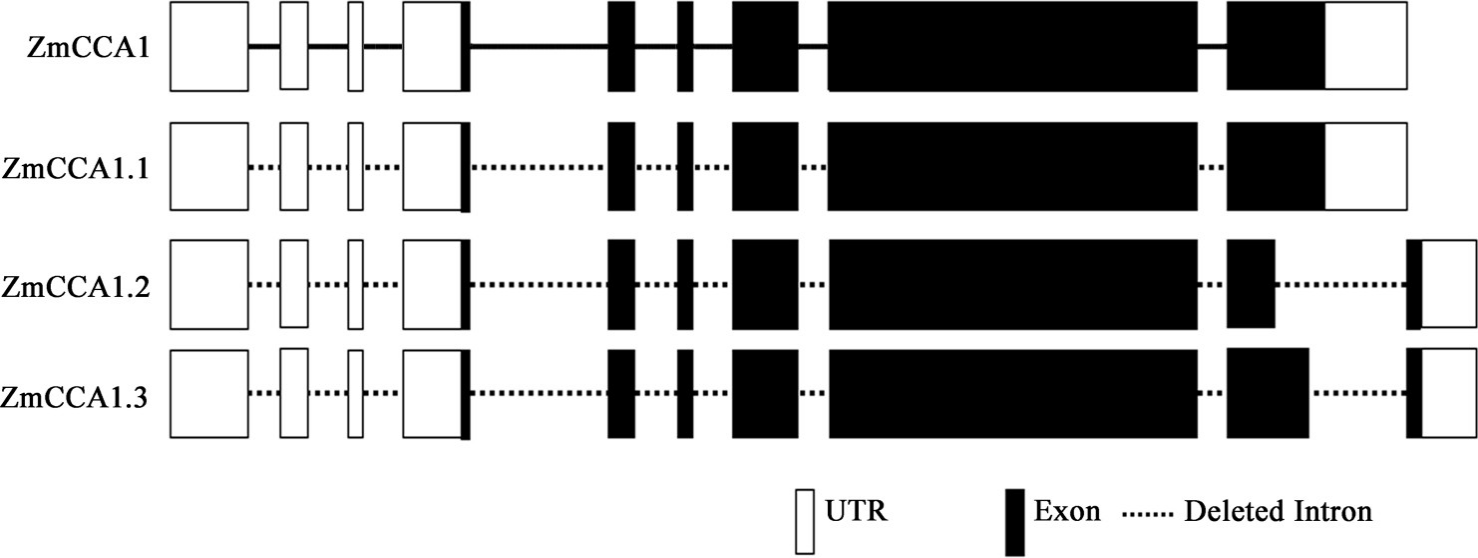
Characterization of three *ZmCCA1* splice variants. Diagrams showing the predicated genomic structures of *ZmCCA1* and three *ZmCCA1* splice variants. White boxes are 3′ and 5′ UTRs. Black boxes depict exons. The deleted introns in three transcripts were represented by dash lines. The introns in ZmCCA1 genome sequence were indicated using solid lines.

In our study, we amplified three PCR products by homologous cloning and the 3′RACE technique. Sequence analysis revealed that *ZmCCA1* was located on chromosome 10 and had three splice variants, i.e., named *ZmCCA1*.*1*, *ZmCCA1*.*2*, and *ZmCCA1*.*3*. *ZmCCA1*.*1* consisted of a 2157 bp ORF encoding a protein of 718 amino acids with a molecular weight of 78.2 kDa, a pI of 6.08, more than 147 bp 5′untranslated region (UTR), and 282 bp 3′UTR, whereas *ZmCCA1*.*2* and *ZmCCA1*.*3* were found to encode proteins of 676 and 709 amino acids with 249 and 87 bp 3′UTR, respectively. Notably, the latter two splice variants had the same length of 5′UTR (>629 bp) (Fig 1, S1–S3 Figs). These results indicated that three splice variants had different structural characteristics.

*ZmCCA1* splice variants are generated by alternative 5′ splice sites, alternative poly (A), and a combination of the above two or more AS types (Fig 1, S2 Fig). We found *ZmCCA1*.*1* encoded authentic proteins, while *ZmCCA1*.*2* and *ZmCCA1*.*3* both encoded truncated proteins. ZmCCA1.1, ZmCCA1.2, and ZmCCA1.3 shared 99.7%, 92.3%, and 97.8% identity with ZmCCA1, respectively. Moreover, we also showed that the N-terminus of the three ZmCCA1 protein isoforms was highly conserved while the C-terminus differed, indicating that individual splice variant potentially possess distinctive functions (S3 Fig).

### Expression of the three *ZmCCA1* splice variants was affected by photoperiod and tissue type

**Fig 2:**
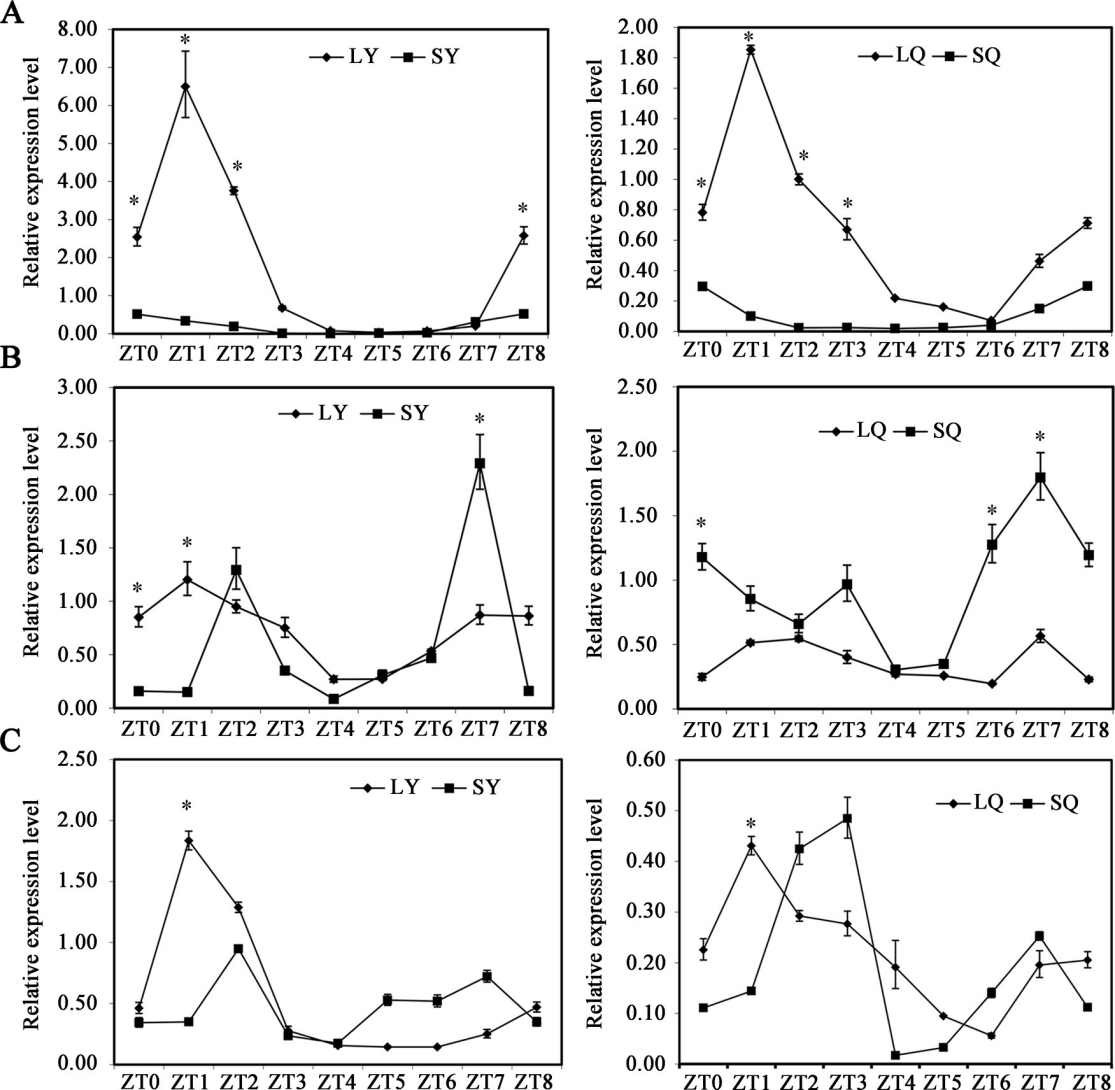
Effects of photoperiod and tissue types on the alternative splicing of *ZmCCA1* in maize. The plant seedlings at five fully-expanded leaf stage on either LDs or SDs were harvested at the indicated ZT points of the extraction of total RNA samples. Relative expression level of *ZmCCA1*.*1* (A), *ZmCCA1*.*2* (B), and *ZmCCA1*.*3* (C) in leaves and leaf sheaths were determined by qRT-PCR. At ZT2, the expression of plant seedlings in the leaf sheaths under LD condition, the leaves under LD condition, and the leaves under SD condition were set to 1 in A, B, and C, respectively. LD, long day conditions; SD, short day conditions; Q, leaf sheaths; Y, leave; ZT, Zeitgeber time. Biological triplicates were averaged. Bars indicate the standard error of the mean. Significant differences between normal and drought treatment condition were assessed using Student’s t-test; * *P* < 0.05.

The expression level of *ZmCCA1* is affected by photoperiod [21]. Moreover, CML288 is much sensitive to night break (NB) treatment under SDs at the five-leaf stage [22, 23]. In this study, we quantified the expression levels of three *ZmCCA1* splice variants in leaves and leaf sheaths at the five fully expanded leaf stage under SD and LD conditions. *ZmCCA1*.*1* transcript levels in leaves and leaf sheaths showed a rhythmic pattern under SDs and LDs and peaked at ZT0 under SDs and at ZT1 under LDs (Fig 2A). *ZmCCA1*.*2* was also expressed rhythmically in leaves under LDs and in leaf sheaths under SDs, respectively peaking at ZT1 and ZT7; transcript levels of *ZmCCA1*.*2* in leaves under SDs or in leaf sheaths under LDs, however, peaked at both ZT2 and ZT7 (Fig 2B). *ZmCCA1*.*3* was expressed rhythmically in leaves and leaf sheaths under LDs, peaking at ZT1. Under SDs, however, transcript levels of *ZmCCA1*.*3* in leaves and leaf sheaths presented two peaks: the former peaking at ZT2 and ZT7 and the latter at ZT3 and ZT7 (Fig 2C). Thus, these results indicate that AS of *ZmCCA1* is affected by photoperiod and tissue type.

*ZmCCA1* transcript levels in leaves and leaf sheaths showed a rhythmic pattern under SDs and LDs and peaked at ZT0 under SDs and at ZT1 under LDs (S4A Fig). At the corresponding peak expression of *ZmCCA1* in leaves and leaf sheaths under SDs or LDs, we compared the expression levels of three *ZmCCA1* splice variants. The results showed that expression levels of *ZmCCA1*.*1* in leaves and leaf sheaths were significantly higher under SDs or LDs, followed by *ZmCCA1*.*2* and *ZmCCA1*.*3*, respectively. Moreover, expression levels of the three *ZmCCA1* splice variants in leaves were higher than those in leaf sheaths under SDs or LDs except for *ZmCCA1*.*2* under SDs (S4B and S4C Fig).

### Expression of three *ZmCCA1* splice variants under drought stress in maize

**Fig 3:**
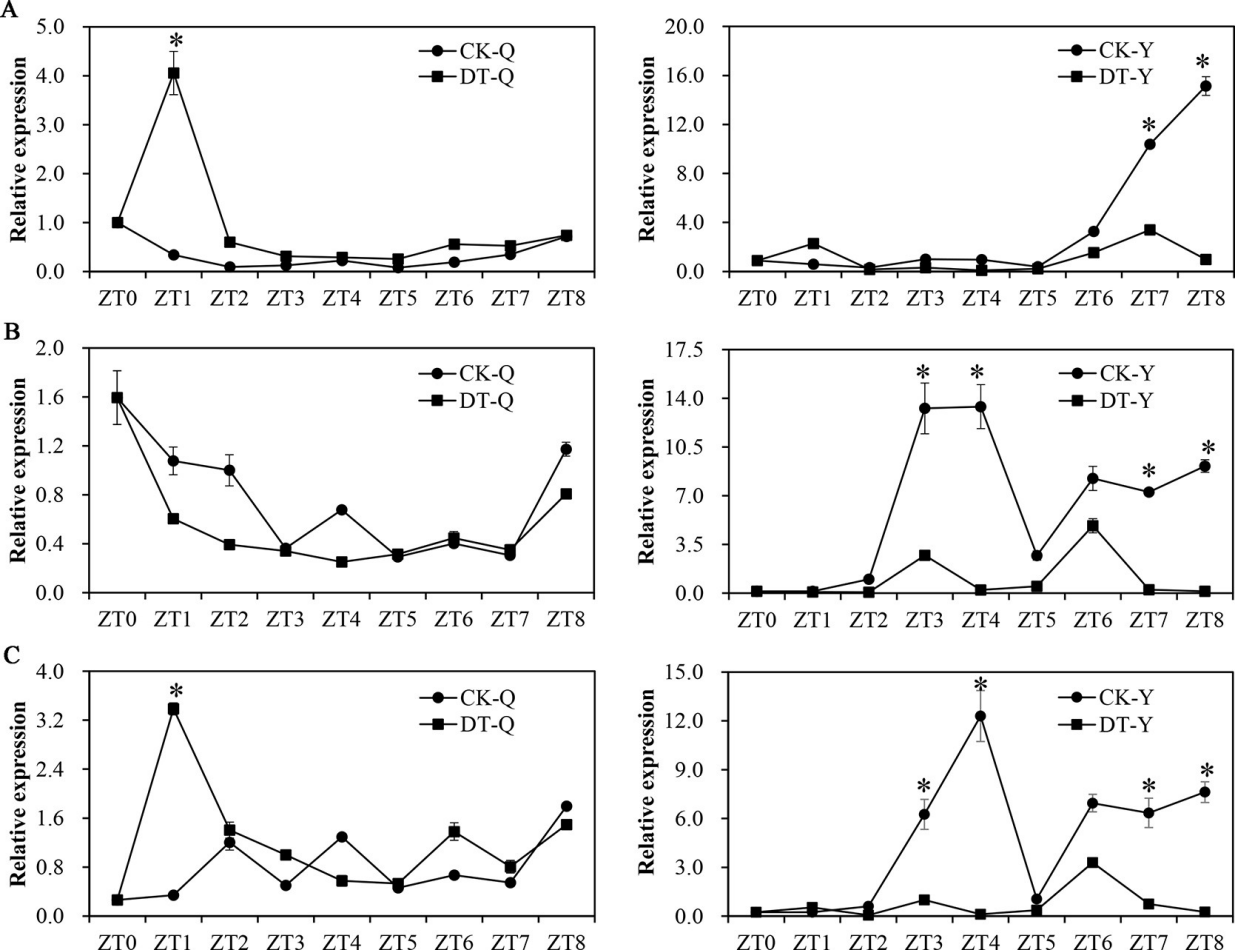
Effects of drought stress on the alternative splicing of *ZmCCA1* in maize. Expression level of *ZmCCA1*.*1* (A), *ZmCCA1*.*2* (B), and *ZmCCA1*.*3* (C) in leaves and leaf sheaths at the five-fully expanded leaf stage after 20% PEG-6000 treatment under continuous light conditions were determined by qRT-PCR. The leaf sheath of seedlings under normal growth condition at ZT0 was considered as 1. Samples were harvested, starting at ZT0 with 3 h intervals for 1 day. CK, seedling grown under normal condition; DT, seedlings grown under 20% PEG-6000 treatment; Q, leaf sheaths; Y, leaves; ZT, Zeitgeber time. Biological triplicates were averaged. Bars indicate the standard error of the mean. Significant differences between normal and drought treatment condition were assessed using Student’s t-test; * *P* < 0.05.

To verify the linkage between AS of *ZmCCA1* and environmental stress responses in maize, CML288 seedlings were treated with 20% PEG-6000 for 1 day at the five fully expanded leaf stage. The RWC of maize leaves grown under normal condition (85.7%) was significantly higher than that under drought stress (57.3%) leaves after 1 day of drought stress (*P* < 0.01) (S5 Fig). After 20% PEG-6000 treatment, compared with the untreated control, expression levels of *ZmCCA1*.*1* first increased in the leaves and leaf sheaths at ZT1, and then the expression in leaf sheath remained continually higher under drought stress; however, in leaves, *ZmCCA1*.*1* showed relatively higher expression level under normal conditions. The expression of *ZmCCA1*.*3* in leaves first increased, then maintained lower levels; however, the expression of *ZmCCA1*.*3* in leaf sheaths first increased, then decreased slowly. Interestingly, transcript levels of *ZmCCA1*.*2* in leaves and leaf sheaths were always lower under drought than that in untreated seedlings (Fig 3). Together, these results indicate that AS of *ZmCCA1* is suppressed under drought stress and that *ZmCCA1*.*1* and *ZmCCA1*.*3* are both involved in drought stress regulation of maize, with *ZmCCA1*.*1* potentially playing a major role. *ZmCCA1*.*2* does not appear to be involved in the drought stress response of maize.

### Overexpression of *ZmCCA1*.*1* and *ZmCCA1*.*3* enhanced tolerance towards drought stress *in transgenic A*. *thaliana*

**Fig 4:**
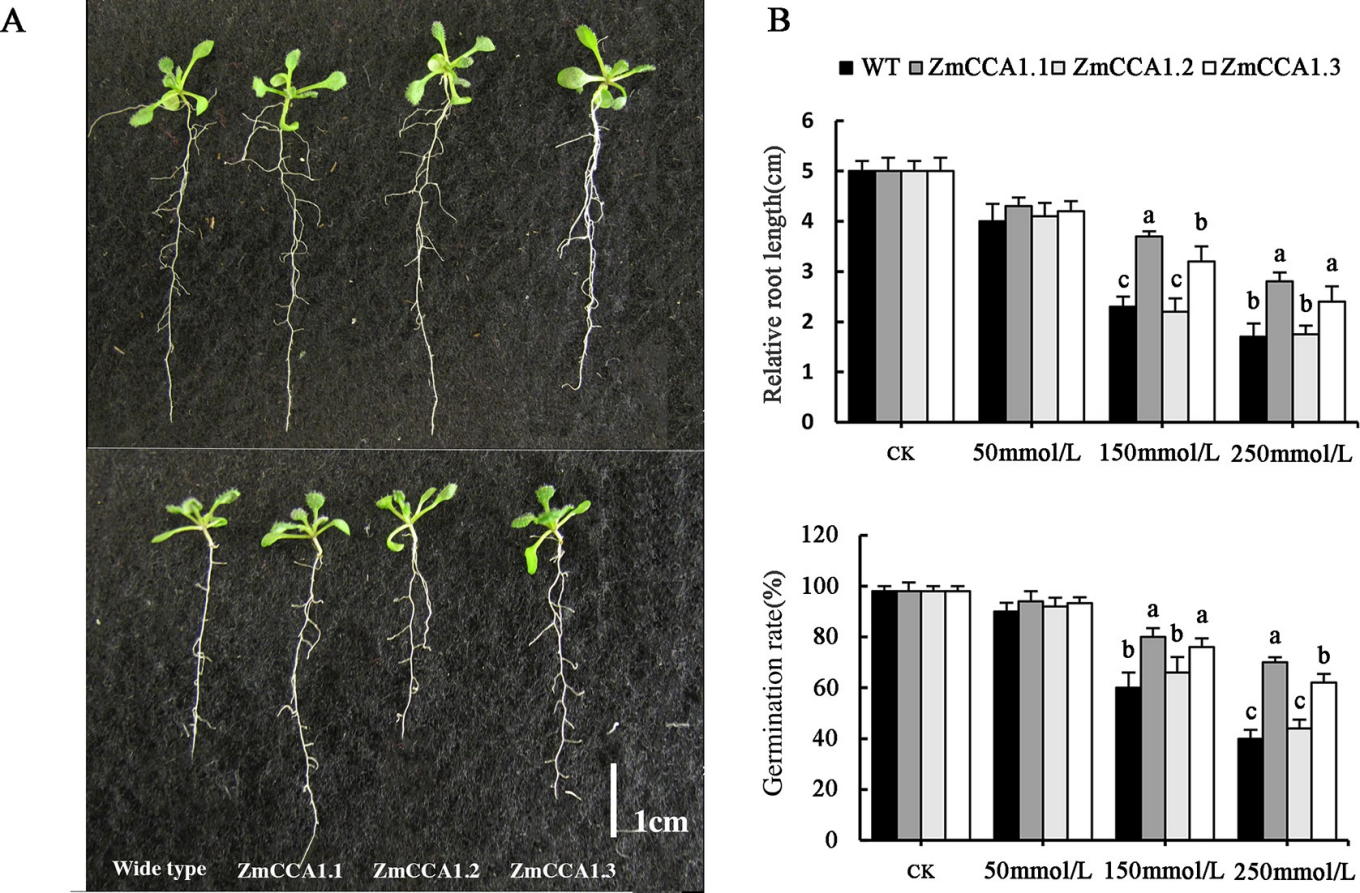
Phenotype analysis of transgenic *A*. *thaliana* lines under drought stress for 2 weeks under LDs. (A) Representative images of 14-day-old transgenic *A*. *thaliana* overexpression lines and wild-type (WT) seedlings under normal (upper panel) and 150 mmol/L mannitol treatment condition (lower panel). (B) Relative root lengths and germination rates of WT and transgenic *A*. *thaliana* overexpression lines under 0–250 mmol/L mannitol. Vertical bars represent standard deviations from the mean. CK, seedlings grown under normal condition. Scale bars in (A) represent 0.5 cm. The different letters (a, b, and c) represent significant differences by ANOVA (*P* < 0.05).

Numerous MYB transcription factors (TFs) have been found to function in cell development and cycling, hormone synthesis, primary and secondary metabolism, and various biotic and abiotic stresses [24–27]. To further determine whether overexpression of *ZmCCA1* splice variants affected tolerance to drought stress in transgenic *Arabidopsis*, we first generated transgenic *A*. *thaliana* plants with overexpression of *ZmCCA1*.*1*, *ZmCCA1*.*2*, and *ZmCCA1*.*3*, which were expressed 15 times higher than in WTs (S6 Fig). Under 50 mmol/L mannitol, the relative primary root lengths of *35S*:*ZmCCA1*.*1*, *35S*:*ZmCCA1*.*2*, and *35S*:*ZmCCA1*.*3* were 12.5%, 2.5%, and 7.5% longer than that of WT plants, respectively, and relative germination rates increased by 3%, 1.6%, and 1.9%, respectively. Under 150 mmol/L mannitol, compared with WT, relative root lengths of transgenic plants were elevated by 39.2%, 7.1%, and 32.1%, respectively, and relative germination rates were 20%, 5%, and 16% higher, respectively. We also found significantly more fibrous roots in *ZmCCA1*.*1* and *ZmCCA1*.*3* transgenic plants than in WT plants under 150 mmol/L mannitol (Fig 4A). Finally, under 250 mmol/L mannitol, relative root lengths were 52.3%, 9.5%, and 33.3% longer than WT, respectively, and relative germination rates were 31%, 5%, and 23% higher, respectively. However, under normal growth conditions, root lengths and germination rates of transgenic *A*. *thaliana* were all consistent with WT (Fig 4B) while the growth of WT *A*. *thaliana* was severely affected under 250 mmol/L mannitol. These results suggest that *ZmCCA1*.*1* may be more effective than *ZmCCA1*.*3* in improving the tolerance of transgenic *A*. *thaliana* plants to drought stress, especially under severe drought conditions, while *ZmCCA1*.*2* may have only a limited effect on increasing tolerance to drought stress in transgenic *A*. *thaliana*.

### Evolutionary analysis of *CCA1* genes and their AS events

**Fig 5:**
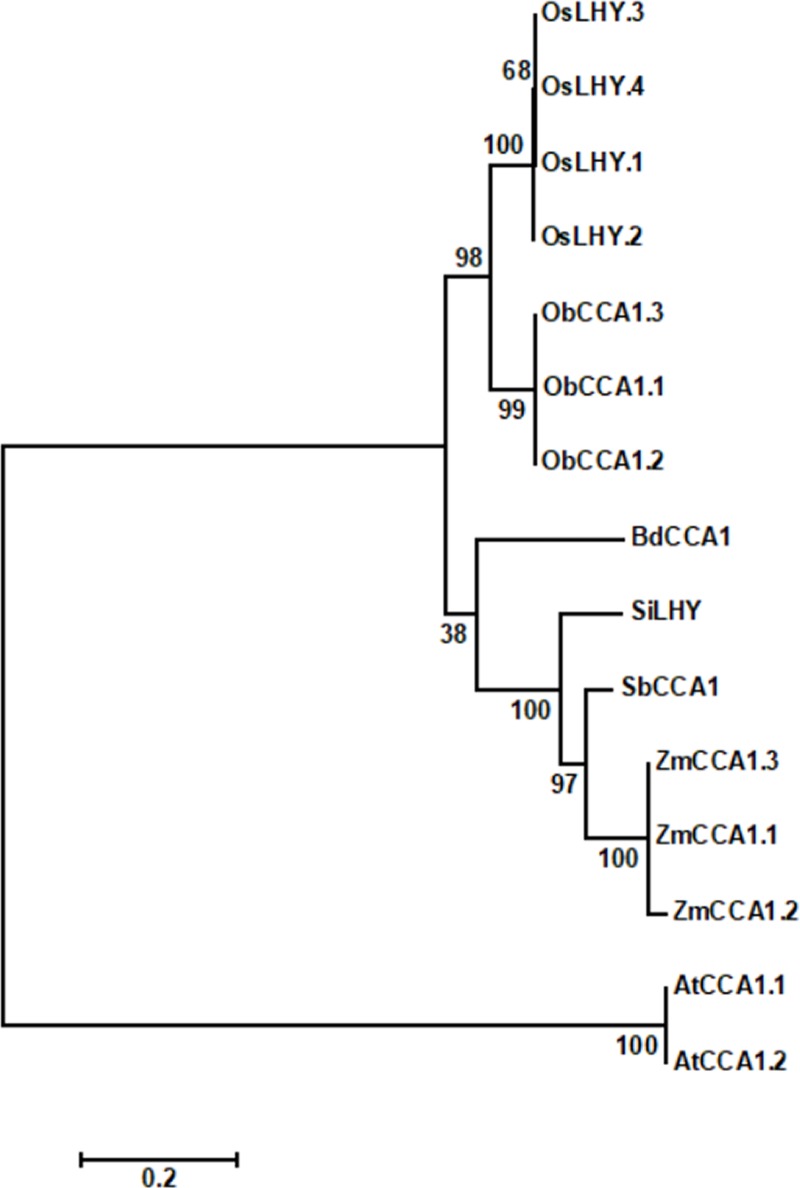
Phylogenetic analysis of CML288 *ZmCCA1* splice variants and related genes. Protein sequences were aligned by DNAMAN version 9.0. The phylogenetic tree was constructed using the maximum-likelihood method with full-length protein sequences. Species names and accession numbers are indicated: *Zea mays*, ZmCCA1 (ACG26686); *A*. *thaliana*, AtCCA1.1 (NP_850460), AtCCA1.2 (NP_001318437); *Sorghum bicolor*, SbCCA1 (LOC8080836); *Setaria italica*, SiLHY (LOC101764638); *Oryza sativa Japonica Group*, OsLHY.1 (XP_015649835), OsLHY.2 (XP_015649844), OsLHY.3 (XP_015649846), OsLHY.4 (XP_015649847); *Oryza brachyantha*, ObCCA1.1 (XP_006659138), ObCCA1.2 (XP_006659145), ObCCA1.3 (XP_015695906); *Brachypodium distachyon*, BdCCA1 (LOC100838310). Scale bar indicates 0.2 of amino acid substitutions per site.

To determine the evolutionary relationship of *ZmCCA1* and those from other species, a phylogenetic tree was constructed using overall protein sequences (Fig 5). *ZmCCA1* splice variants and *ZmCCA1* were located in the same subfamily. Compared with *A*. *thaliana*, a dicotyledonous species, *CCA1* genes were more homologous among the monocotyledon species, especially in C4 plants.

We provided experimental evidence to support the occurrence of three *ZmCCA1* splice variants on chromosome 10 in maize (Fig 5). *AtCCA1* and *OsCCA1* have also been consistently found to undergo AS [28, 29]. To explore whether AS events of *CCA1* genes are conserved across species, we identified *CCA1* homolog genes in several well-annotated genomes (Table 1). We found that there is only one homologous gene in numerous species, producing three splice variants encoding three protein isoforms on chromosome 10 in *Zea mays*, 21 splice variants in *Sorghum bicolor*, 7 splice variants in *Setaria italica*, 12 splice variants in *Oryza sativa Japonica Group*, 9 splice variants in *Oryza brachyantha*, 4 splice variants in *Brachypodium distachyon*, and 3 splice variants in *Arabidopsis*, respectively encoding 1, 1, 4, 3, 1, and 2 protein isoforms. Collectively, these results suggest that AS events of *CCA1* are present in numerous species, but occur with species specificity.

**Table 1:** List of *CCA1* homologous genes and their predicted splice variants in plants.

Organism	Annotated gene number in each species	Chromosome	Locus ID	Number of AS for each gene	Number of protein isoforms
***Zea mays***	1	10	*GRMZM2G474769*	3	3
***Sorghum bicolor***	1	7	*LOC8080836*	21	1
***Setaria italica***	1	6	*LOC101764638*	7	1
***Oryza sativa Japonica Group***	1	8	*LOC4344703*	12	4
***Oryza brachyantha***	1	8	*LOC102717637*	9	3
***Brachypodium distachyon***	1	3	*LOC100838310*	4	1
***Arabidopsis thaliana***	1	2	*AT2G46830*	3	2

Predicted splice variants were collected from the National Center for Biotechnology Information (NCBI; https://www.ncbi.nlm.nih.gov/; NCBI B73-v4 annotation release 101), the Gramene (http://www.gramene.org/), the Maize Genetics and Genomics Database (MaizeGDB; https://www.maizegdb.org/; Zm-B73-REFERENCE-GRAMENE-4.0), the National Center for Biotechnology Information (NCBI; https://www.ncbi.nlm.nih.gov/), and the A. thaliana Information Resource (TAIR, http://www.arabidopsis.org/).

## Discussion

Alternative splicing (AS) is a widespread phenomenon in higher eukaryotes. In *Arabidopsis*, at least 61% of intron-containing genes are AS [30]. In *O*. *sativa*, 33% of all rice genes undergo AS [31]. Approximatively, 55.3% of maize genes may be subjected to AS [32]. In this study, we confirmed by homologous cloning and the 3′RACE technique that *ZmCCA1* undergoes extensive AS, which occurred at alternative 5′ splice site, alternative poly (A), or by a combination of two or more AS types, encoding three protein isoforms, i.e., ZmCCA1.1, ZmCCA1.2, and ZmCCA1.3. AS isoforms were found to be conserved between one maize homolog and its sorghum ortholog in our study, but absent from the second maize homolog. These results may support the published predication that AS isoforms may have been lost after the maize whole genome duplication event [33].

There are two hypotheses about the function of splice variants: i.e., the “noise” hypothesis, and the “regulated unproductive splicing and translation (RUST)” hypothesis. Based on the former hypothesis, NMD-sensitive RNA variants are generated due to splicing error and are finally removed through the NMD pathway [34–36]. However, the later hypothesized that alternative mRNA splicing coupled with NMD is also known to dynamically monitor the abundance of full-size RNA splicing, particularly during various forms of cellular stress [37–39]. Interesting, recent reports have provided an overview that TF genes can generate protein isoforms without specific functional domains by AS, which competitively inhibit the authentic TFs by forming nonfunctional heterodimers [14, 40–42]. In this study, we confirmed that AS of *ZmCCA1* is influenced by tissue type, photoperiod, and drought stress in maize. By constructing the overexpression vector of these three variants, we found that *35S*:*ZmCCA1*.*1* and *35S*:*ZmCCA1*.*3* (Col-0) transgenic plants all showed significantly higher drought tolerance whereas *35S*:*ZmCCA1*.*2* (Col-0) exhibited only slightly higher drought tolerance, indicating that these three splice variants were identified to perform different tolerance abilities in response to drought, and further support that the RUST hypothesis potentially fits well into the AS of *ZmCCA1*.

In addition, AS events of *CCA1* were identified across various species but occurred with species specificity, which helps to further explore the AS function of circadian-associated genes in other plant species. Moreover, AS could be affected by tissue types, photoperiod, and abiotic stress [16, 33, 43–46]. Given that in maize hybrids, CCA1 proteins target thousands of output genes early in the morning, as if the hybrids wake up early to promote photosynthesis, starch metabolism, and biomass accumulation [37–39]; therefore, AS of *ZmCCA1* should play more complex roles than previously expected.

In conclusion, three *ZmCCA1* splice variants, i.e., *ZmCCA1*.*1*, *ZmCCA1*.*2*, and *ZmCCA1*.*3*, were cloned and obtained a highly conserved N-termini and different C-termini, which were influenced by photoperiod, tissue type, and drought stress in maize. Overexpression of these three *ZmCCA1* variants resulted in increased tolerance to drought stress. *ZmCCA1*.*1* may be more effective than *ZmCCA1*.*3* with higher drought tolerance, whereas *ZmCCA1*.*2* performed with a relatively limited effect on increasing tolerance to drought stress. Additionally, AS events of *CCA1* genes may occur with species specificity. Thus, this study lays a basic foundation to understand the function of *ZmCCA1* in drought resistance mediated by AS in maize, and further provides new evidence to link the circadian clock, AS, and abiotic stress.

## Supporting information

S1 Table Primers used in the quantitative real-time RT-PCR analysis. (XLSX)

S1 Fig Multiple sequence alignments of *ZmCCA1* transcript sequences with *ZmCCA1* gDNA (AC215881.5). A large fragment insertion was indicated by “X”.
(TIF)

S2 Fig Multiple sequence alignments of 3′ UTR sequences of *ZmCCA1*. (TIF)

S3 Fig Multiple sequence alignments of the protein sequences of ZmCCA1 splice variants. The MYB domain is marked by a black line.
(TIF)

S4 Fig Relative expression of three *ZmCCA1* splice variants. (A) Relative expression of *ZmCCA1* in leaves and leaf sheaths at the five fully expanded leaf stage under LDs and SDs. Significant differences between leaf sheaths and leaves were assessed using Student’s t-test; * *P* < 0.05. (B, C) Relative expression of three *ZmCCA1* splice variants in leaves and leaf sheaths at the five fully expanded leaf stage at the corresponding peak expression of *ZmCCA1* under LDs (B) and SDs (C). L, LDs; S, SDs; Q, leaf sheaths; Y, leaves. Vertical bars represent standard deviations from the mean.
(TIF)

S5 Fig Relative water loss (RWC) in maize under drought stress compared to the seedlings under normal conditions. **RWC was measured with detached leaves from seedlings.** Significant differences between normal and drought treatments condition were assessed using Student’s t-test; * *P* < 0.05.
(TIF)

S6 Fig Relative expression level of three splice variants (*ZmCCA1*.*1*, *ZmCCA1*.*2*, and *ZmCCA1*.*3*) in transgenic *Arabidopsis*. Significant differences between wild type and three splice variants were assessed using Student’s t-test; ** *P* < 0.01. WT, wild type.
(TIF)
