# Supplementary figures and images for: Alternative splicing of ZmCCA1 mediates drought response in tropical maize
Source: PLoS One. 2019 Jan 30;14(1):e0211623. doi: 10.1371/journal.pone.0211623 (PMC6353190; doi:10.1371/journal.pone.0211623)

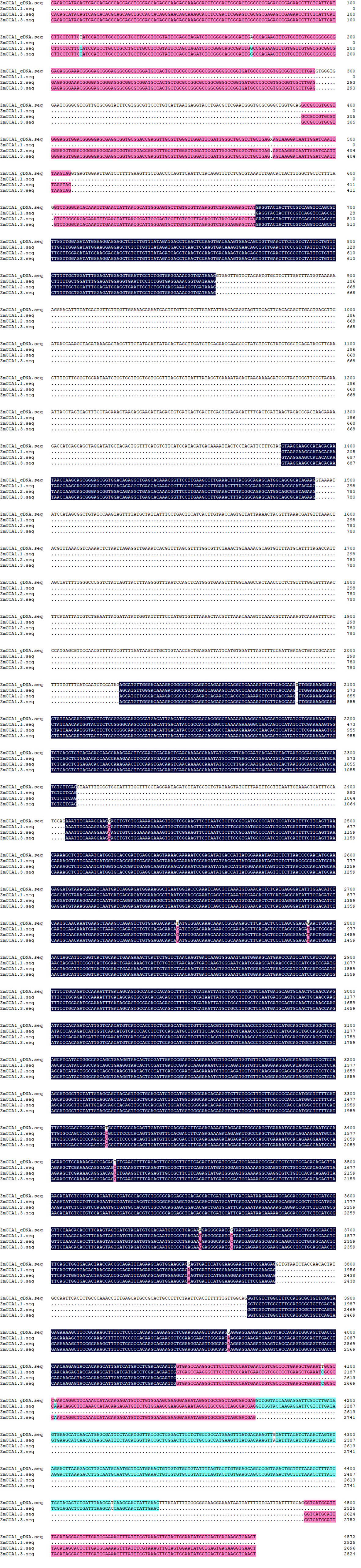

Supplement: S1 Fig — A large fragment insertion was indicated by “X”. (TIF) [file pone.0211623.s002.tif]

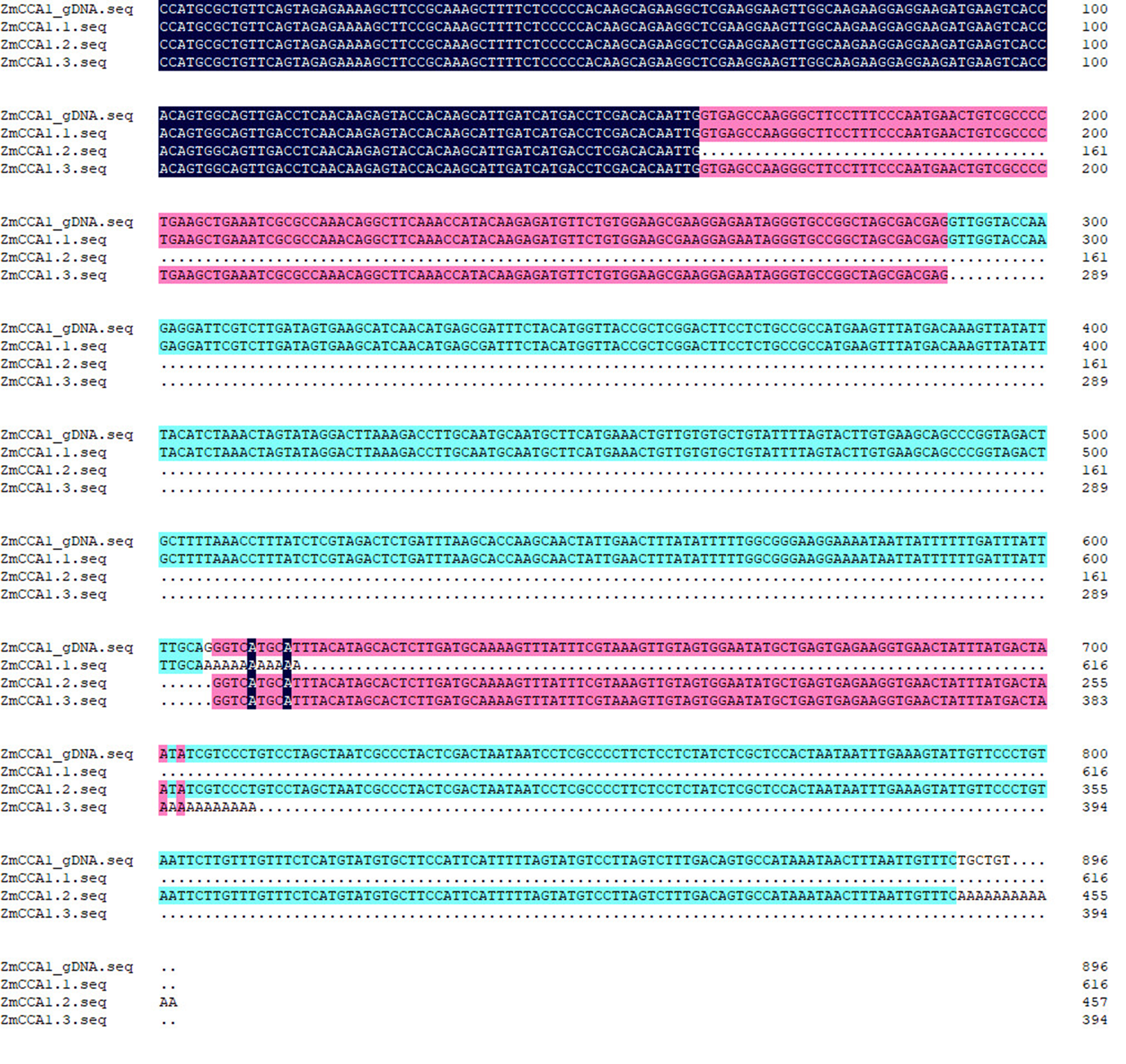

Supplement: S2 Fig — (TIF) [file pone.0211623.s003.tif]

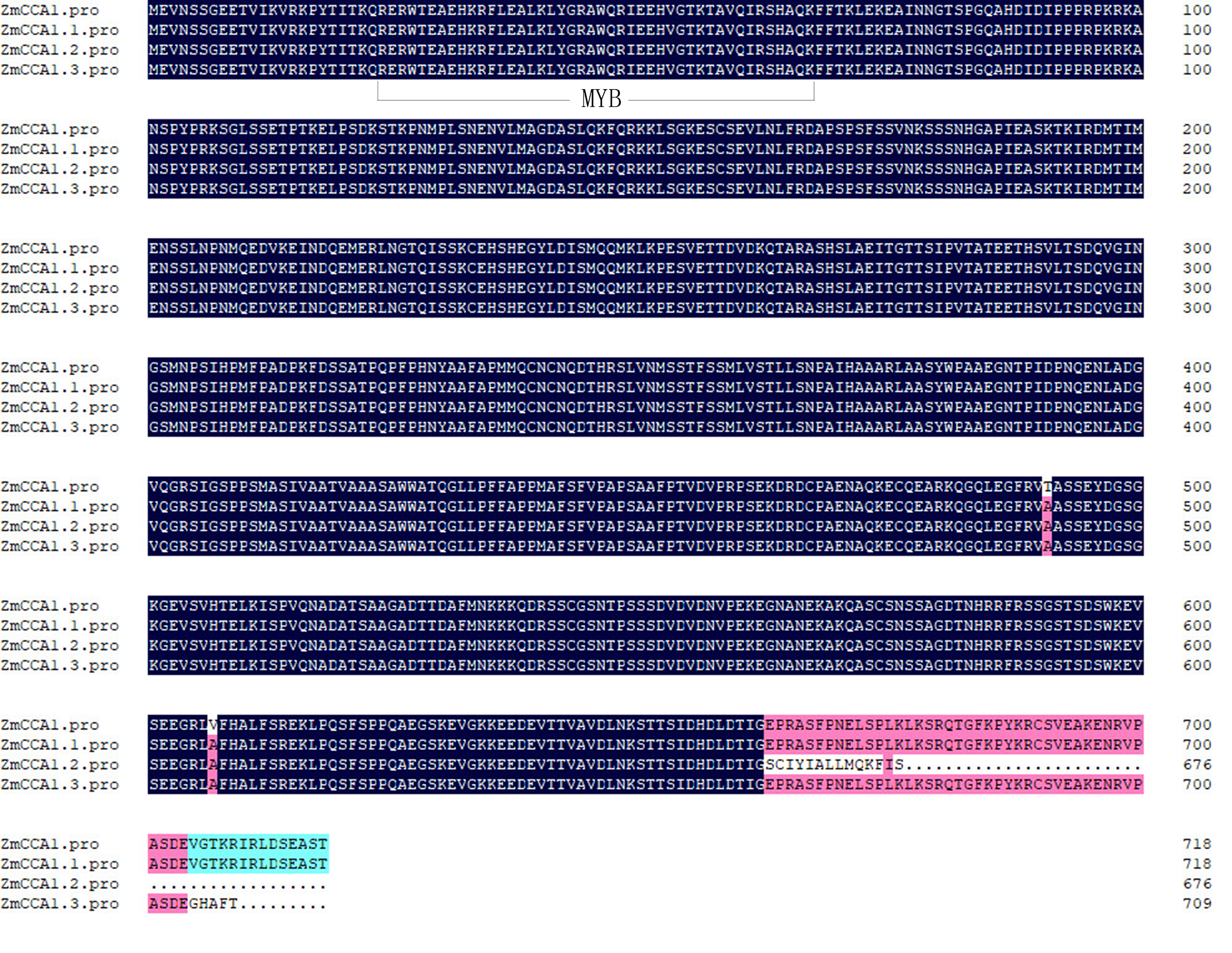

Supplement: S3 Fig — The MYB domain is marked by a black line. (TIF) [file pone.0211623.s004.tif]

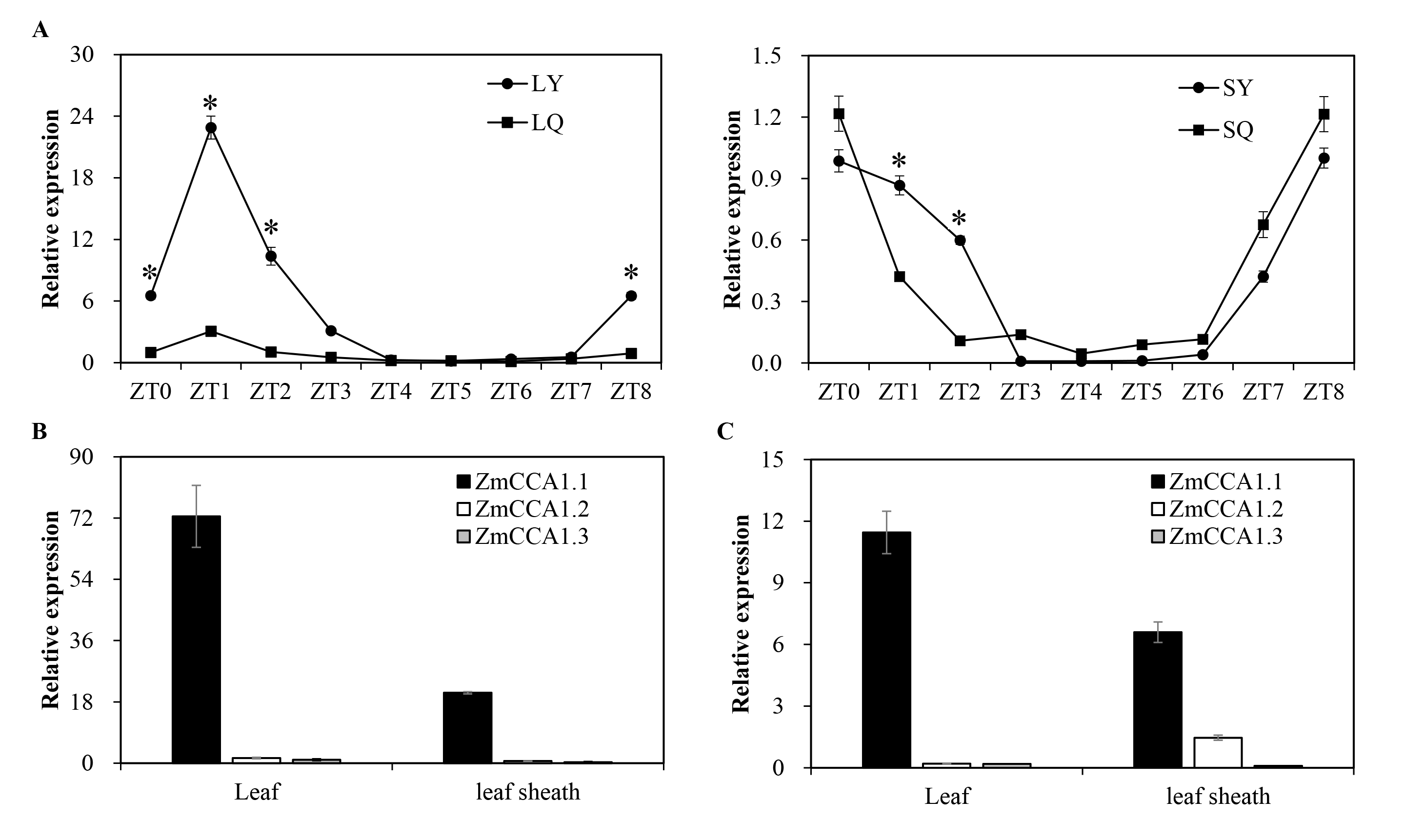

Supplement: S4 Fig — (A) Relative expression of ZmCCA1 in leaves and leaf sheaths at the five fully expanded leaf stage under LDs and SDs. Significant differences between leaf sheaths and leaves were assessed using Student’s t-test; * P < 0.05. (B, C) Relative expression of three ZmCCA1 splice variants in leaves and leaf sheaths at the five fully expanded leaf stage at the corresponding peak expression of ZmCCA1 under LDs (B) and SDs (C). L, LDs; S, SDs; Q, leaf sheaths; Y, leaves. Vertical bars represent standard deviations from the mean. (TIF) [file pone.0211623.s005.tif]

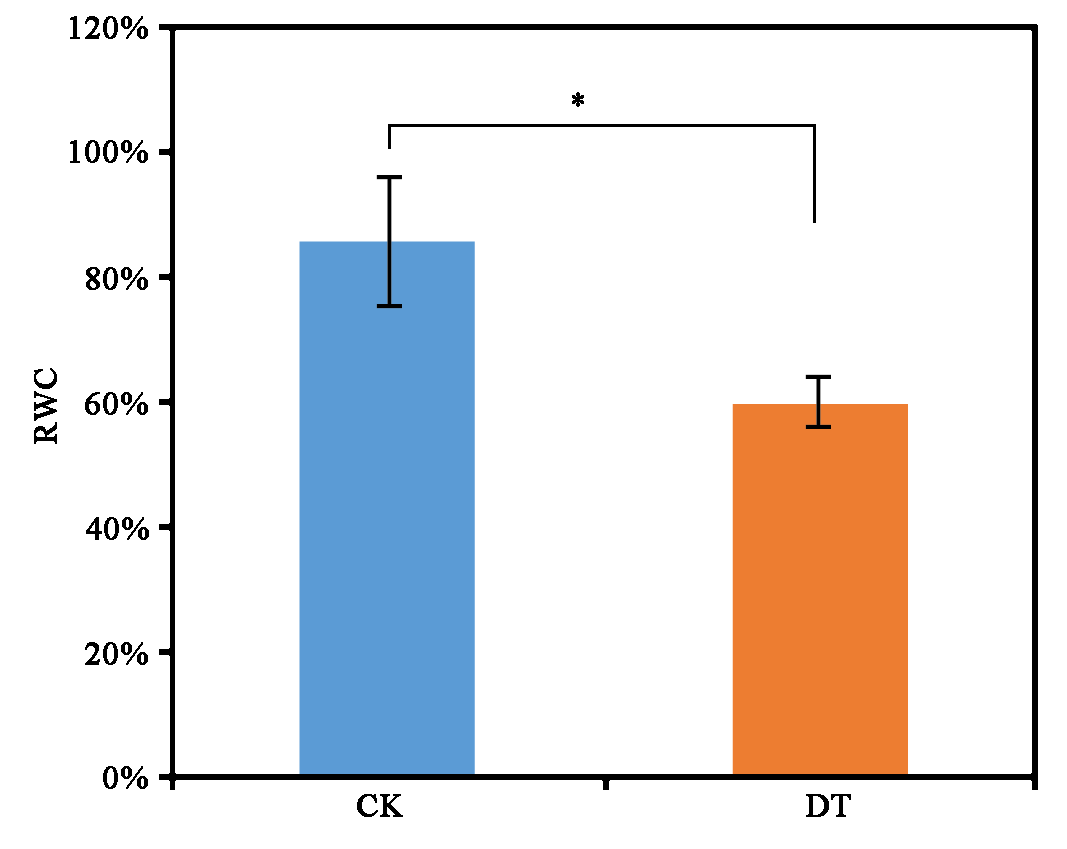

Supplement: S5 Fig — RWC was measured with detached leaves from seedlings. Significant differences between normal and drought treatments condition were assessed using Student’s t-test; * P < 0.05. (TIF) [file pone.0211623.s006.tif]

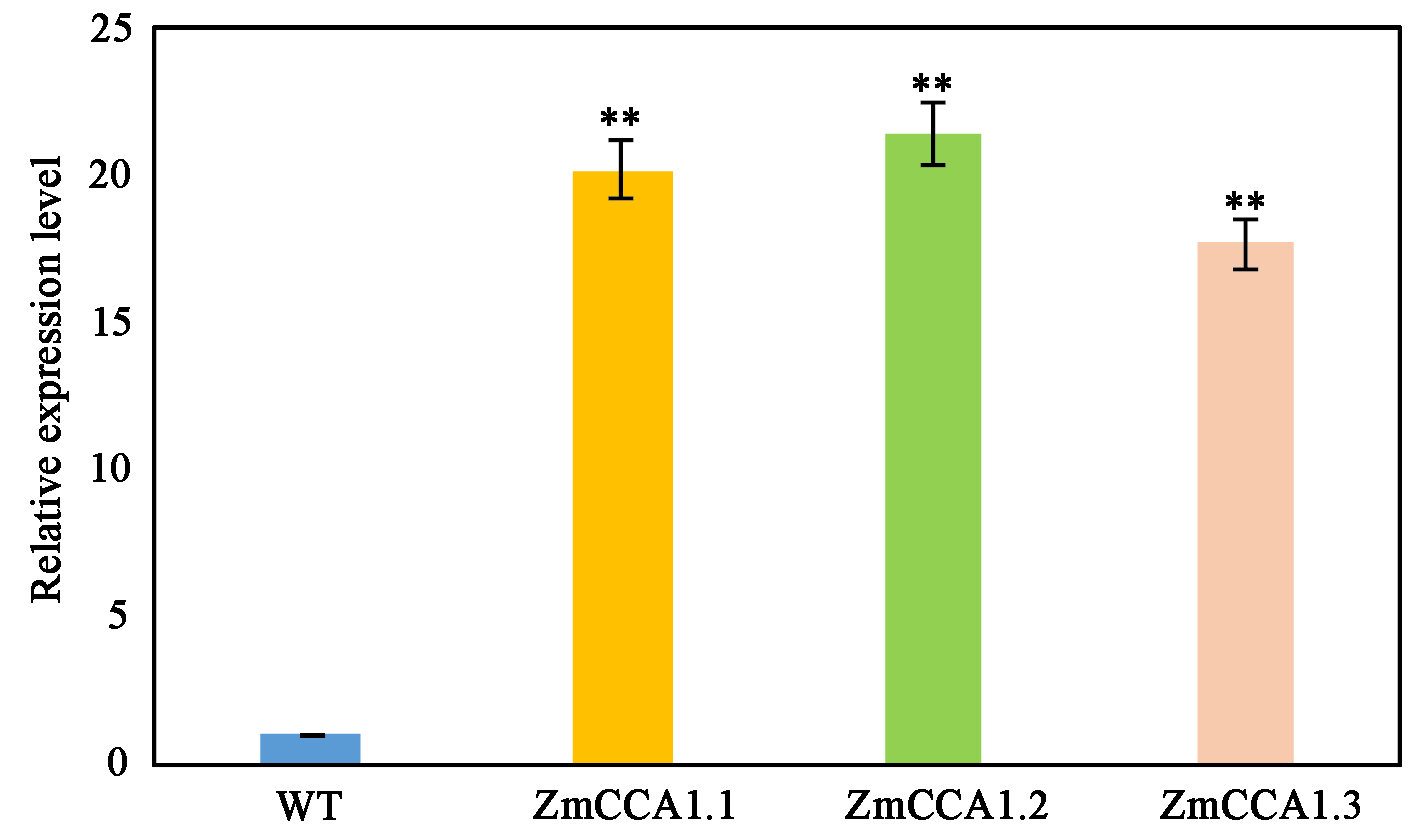

Supplement: S6 Fig — Significant differences between wild type and three splice variants were assessed using Student’s t-test; ** P < 0.01. WT, wild type. (TIF) [file pone.0211623.s007.tif]
